# Design and use of a wireless temperature measurement network system integrating artificial intelligence and blockchain in electrical power engineering

**DOI:** 10.1371/journal.pone.0296398

**Published:** 2024-01-02

**Authors:** Dianshuai Dong, Hongliang Feng

**Affiliations:** Wuxi Guangying Group Co., Ltd, Wuxi City, China; TU Wien: Technische Universitat Wien, AUSTRIA

## Abstract

This work aims to investigate the potential fire hazard stemming from the overheating of power equipment. The advent of the artificial intelligence era has facilitated the fusion of blockchain and Internet of Things (IoT) technologies. This work delves into the technical standards for IoT equipment monitoring and smart grid communication, and the IoT environment of power grid equipment. This work introduces a temperature monitoring network tailored for IoT wireless power equipment suitable for the power environment, and conducts system debugging in the power laboratory. The findings affirm that the temperature out-of-limit alarm testing has met the required criteria, confirming the system’s ability to issue timely warnings when temperatures breach a predefined threshold, effectively avoiding high-temperature misfires. This work fully harnesses the secure and user-friendly operation of smart blockchain and the wireless sensing technology of the IoT to realize online monitoring and remote temperature measurement of the power system. It can effectively prevent equipment from overheating and damage, and promote the development of equipment condition monitoring technology in electric power engineering.

## 1. Introduction

The equipment failure rate has increased yearly with the rapid progress of modern electric power engineering. The timeline for workers to remove faults is lengthened, which leads to the fire caused by the untimely handling of power equipment. Thereby, the temperature monitoring of power equipment becomes increasingly important. Recognizing that there is a window of time between abnormal heating and equipment failure that can lead to accidents, the early detection of abnormalities and the implementation of corresponding measures can significantly reduce or even eliminate such power-related accidents [1]. Consequently, conducting online temperature monitoring of operational power equipment, especially high-voltage equipment, has become an urgent imperative for ensuring the safety of the power system. On this basis, this work introduces wireless sensor temperature measurement technology capable of real-time temperature data monitoring through remote sensing devices. This technology enables early warnings when power equipment temperatures approach critical thresholds, thus effectively avoiding the occurrence of fire [2].

The rapid advancement of Internet of Things (IoT) technology has paved the way for the development of wireless temperature measurement systems for the power industry. These systems can establish connectivity between the Internet and articles through information-sensing devices such as radio frequency identification, global positioning systems, laser scanners and infrared sensors. This connectivity enables seamless information exchange and communication, facilitating intelligent identification, tracking, monitoring, and management of data of the power’s temperature measurement system [3]. Ebenezer et al. proposed a next-generation public network strategy that harnessed the unused TV white spaces spectrum. This strategy aggregated idle TV white space spectrum into usable channels, differentiating the next-generation public network from traditional power networks in terms of security, reliability, self-awareness, and cross-layer compatibility [4]. Under the Internet environment, implementing suitable information security guarantee mechanisms empowers personalized, safe, and controllable real-time online monitoring, remote maintenance, online upgrading and other management and service functions to integrate management and control [5]. As the electricity demand continues to rise, the power’s wireless temperature measurement system based on IoT technology is poised to garner increasing attention in the future.

Blockchain is a disruptive and innovative technology that aligns closely with the strategic goals of the national power grid. It addresses issues such as data interoperability, device security, and multi-party collaboration in the construction of the power IoT [6]. The introduction of artificial intelligence (AI) further enhances the application of blockchain in power IoT, facilitating intelligent analysis and prediction to optimize energy management and ensure data security and device monitoring. This amalgamation of blockchain’s decentralized and tamper-proof features paves the way for the creation of an intelligent and dependable temperature measurement system within the power IoT, propelling advancements in power IoT technology. The integration of AI also accelerates the research and application of cutting-edge blockchain technologies, enhancing the brand value of the national power grid and driving the transformation and sustainable development of the power industry [7].

Based on the above problems, this work initially delves into the scheme design of power’s wireless temperature measurement system, and summarizes the existing temperature monitoring technology for power equipment. Meanwhile, massive technical specifications related to electric power engineering have been consulted to analyze the current temperature monitoring of power equipment and summarize the technical requirements of wireless temperature measurement system design. This work effectively combines IoT wireless sensors with a power temperature measurement system and introduces AI and blockchain technology to design an intelligent wireless temperature measurement system tailored for power engineering. By leveraging AI technology, the system can intelligently analyze temperature data from power equipment and achieve temperature warnings and anomaly detection, thereby enhancing equipment failure identification and prevention. Meanwhile, the introduction of blockchain technology ensures data security, immutability, and traceability, enabling trusted transmission and storage of temperature data from power equipment. The harmonious fusion of AI and blockchain technologies in the wireless temperature measurement system for power engineering bolsters the system’s intelligence, data credibility, and device security. It ultimately promotes the modernization and sustainable development of the power industry.

This work has made significant theoretical and practical contributions to the development of power engineering equipment’s condition monitoring technology. First, it designs a temperature monitoring network suitable for IoT wireless power equipment in a power environment. This network incorporates smart blockchain, IoT wireless sensor technology, and the temperature measurement requirements of smart grid equipment. It enables online monitoring and remote temperature measurement within the power system, effectively preventing equipment overheating and damage. Next, this work introduces a proactive protective mechanism based on temperature out-of-limit alarms, capable of issuing timely warnings when temperatures reach predefined thresholds. This mechanism effectively prevents high-temperature fires and demonstrates the system’s feasibility and practicality. Additionally, this work adopts AI technology to design intelligent temperature data analysis for power equipment, facilitating temperature warnings and anomaly detection, thereby enhancing the identification and prevention of equipment failures and ensuring the safe operation of power equipment. The incorporation of blockchain technology ensures data security, immutability, and traceability, enabling trustworthy transmission and storage of power equipment temperature data, thereby bolstering data credibility and device security within the system. Finally, this work innovatively integrates AI, blockchain, and IoT technology into the wireless temperature measurement system for power engineering, driving the modernization, transformation, and sustainable development of the power industry. This work delves deeply into the design of the power’s wireless temperature measurement system, summarizes existing temperature monitoring technologies for power equipment, and provides valuable insights and references for future related studies.

## 2. Literature review

Due to the increasing use of power resources in the industry and people’s lives, worldwide research on electric power engineering safety is also increasingly valued at this stage, especially the research on power temperature testing and monitoring technology [8]. Laghari et al. viewed IoT as a system that interconnectd computing devices, machinery, digital machines, and personal devices with unique system identifiers, facilitating data transfer between devices without human intervention [9]. They introduced a modular architecture supporting blockchain sawtooth and a data structure that enabled smart contracts to ensure streamlined industrial node transactions and content broadcasting [10]. Waqas et al. primarily focused on network security issues within the IoT, particularly in the context of zombie network attacks. They investigated Botnet Intrusion Detection Systems (B-IDS), Distributed Denial of Service (DDOS) attacks, and network security when occurring malicious software threats. The findings highlighted the effectiveness and reliability of detecting zombie network attacks [11]. Khan et al. delved into innovative research concerning IoT and Wireless Sensor Network (WSN) systems, discussing the wide-ranging applications of multimedia systems in areas such as edge computing, healthcare, live streaming, and agricultural technology [12]. Huang et al. proposed the application of a Generative Adversarial Network (GAN) model based on intelligent data analysis for music emotion recognition in the context of the IoT. This model effectively identified music emotions with an accuracy exceeding 87%, surpassing traditional music emotion recognition methods [13]. Nazir et al. comprehensively reviewed wireless communication architectures and protocols, analyzed the security threats faced by wireless local networks and proposed solutions to counter these threats [14]. Khan et al. introduced the Blockchain-based Healthcare Industrial Internet of Things (BHIIoT), a blockchain distributed ledger technology, which was employed to secure electronic medical data. They utilized the NuCypher threshold re-encryption mechanism for data encryption [15]. Khan et al. combined the generative capabilities of the GAN and the processing capabilities of fuzzy logic to effectively compress and encode multimedia streaming data [16].

Jiang used IoT technology and Zigbee transmission technology to design the wireless temperature measurement system of power cables. This innovative design realized the temperature data acquisition and transmission in the power cable. Then, temperature measurement software was employed to monitor the information remotely. The entire system exhibited commendable reliability and robust stability, signifying its pivotal role in the application of temperature measurement in electric power engineering [17]. Emmanuel et al. introduced an optimized beamforming strategy that leveraged a dual combination of simulated beamforming and hybrid analog/digital beamforming to counteract additional propagation losses in outdoor millimeter-wave environments [18]. Huang et al. studied the application of an infrared temperature monitoring system in electric power temperature measurement. They employed fiber grating sensors to track the temperature changes of electrical equipment in real time, and provide necessary alarm signals to effectively prevent fire in the power system [19].

There have been some advancements in the research of combining AI with blockchain in wireless temperature measurement technology for the power sector. Several noteworthy research examples illustrate the current landscape. Dhar proposed the possibility of leveraging AI technology to diagnose and predict faults using power equipment temperature data. The establishment of machine learning models and deep learning algorithms enabled intelligent equipment status analysis. It could facilitate early fault prediction and the implementation of corresponding maintenance and repair measures to minimize downtime and losses [20]. Mohanta believed that combining AI and blockchain technology could enable real-time monitoring and optimization of power systems. By employing intelligent algorithms to analyze temperature data in real-time, equipment operating parameters could be adjusted, leading to enhanced energy utilization efficiency, improved system stability, and increased reliability [21]. Issa et al. developed a blockchain-based federated learning approach to safeguard data privacy and security within IoT systems [22]. Li et al. introduced a private three-layer local blockchain architecture known as Three-Layer Blockchain (TBchain) to address security and privacy issues stemming from the centralization of existing IoT infrastructures. This architecture achieved data security and privacy protection by splitting and locking some transactions from the public blockchain into a higher-level TBchain [23]. Ferrag and Shu summarized existing research on blockchain security in IoT networks, and reviewed various blockchain-based security and privacy systems for various IoT applications [24]. Rani et al. leveraged the strengths of software-defined networking and blockchain technology to develop an innovative adaptive network infrastructure tailored for smart cities [25]. Ali et al. proposed a novel approach that combined homomorphic encryption technology with blockchain to enhance privacy protection in IoT-based healthcare applications [26].

To sum up, relevant scholars have researched the temperature measurement technology of electric power engineering, proving that the application of wireless sensor technology in electric power temperature measurement has great prospects. However, previous research has some limitations. For example, in the context of IoT system connectivity and data transmission, existing studies have not fully explored the potential of blockchain and have failed to provide comprehensive security measures. While progress has been made in detecting zombie network attacks in IoT network security research, data privacy protection remains somewhat lacking. In the realm of multimedia systems and healthcare applications, although there has been some research advancement, more in-depth exploration is still required for effective control of data privacy protection. Regarding intelligent data analysis, despite the application of relevant models, further research is needed to enhance the robustness and real-time capabilities of these models. In the domain of temperature monitoring solutions for electric power engineering, existing research still has certain limitations in real-time monitoring and remote data transmission. For the limitations and shortcomings of the existing research, this work aims to comprehensively harness AI and blockchain technology to enhance data privacy protection and security control in temperature monitoring solutions for electric power engineering within the IoT. This approach can provide a more comprehensive and reliable solution. Simultaneously, incorporating intelligent data analysis technology can improve the recognition and predictive capabilities for temperature variations in power equipment, offering a more efficient and intelligent approach to power engineering safety management. By bridging the gaps in existing research, this work is committed to achieving further breakthroughs in the design and application of temperature monitoring solutions for electric power engineering.

## 3. Materials and methods

### 3.1 IoT technology

**Fig 1:**
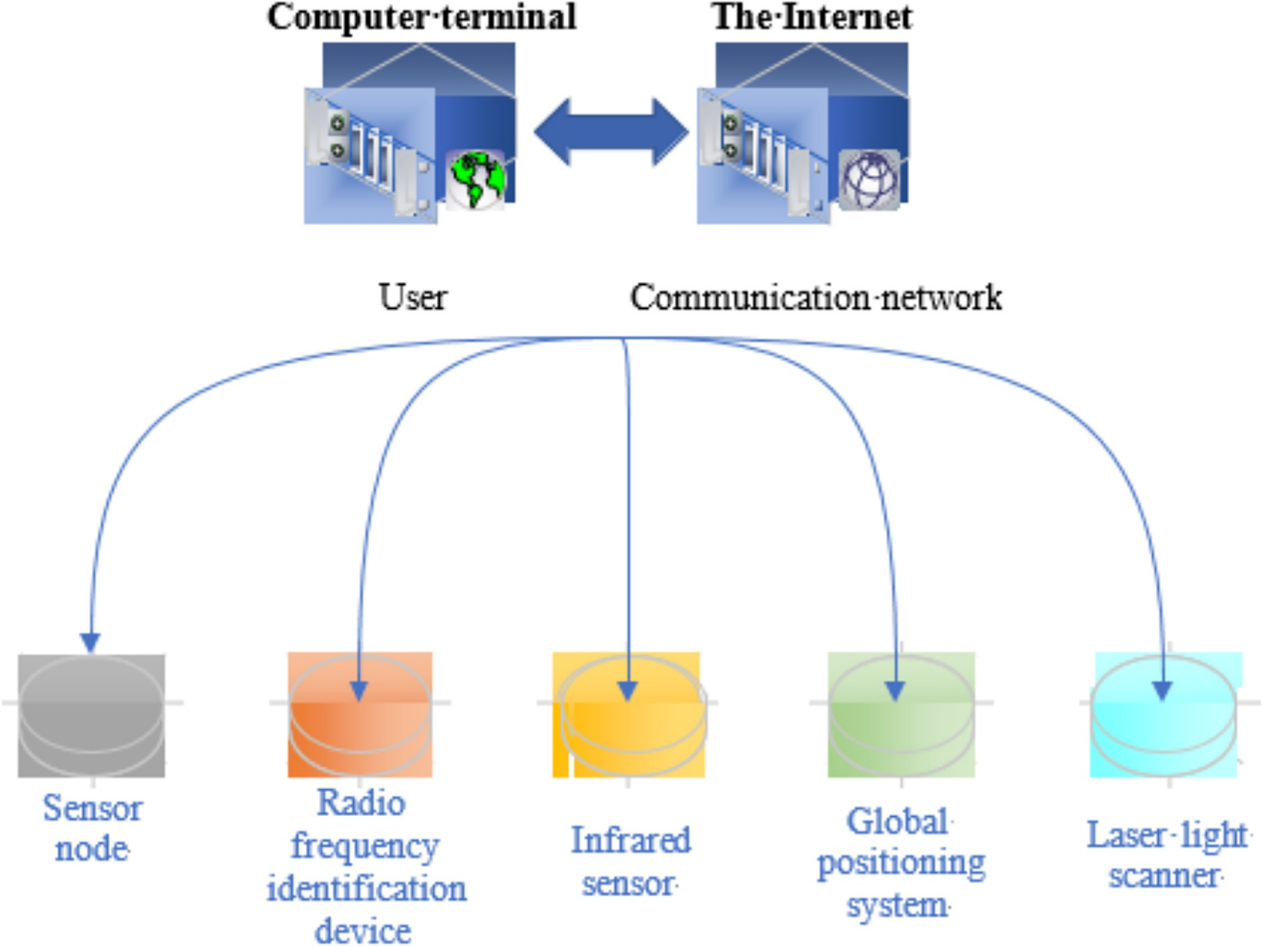
IoT definition.

The concept of IoT was initially proposed in 1999, primarily to manage electronic product codes within the supply chain. However, over the years, as technology evolves and applications develop, the scope and utilization of IoT technology expanded significantly. Fig 1 displays the details:

IoT is defined as a network that establishes connections between various objects and the Internet through information sensing equipment. This equipment includes technologies such as Radio Frequency Identification (RFID), infrared sensors, and global positioning systems, all operating according to agreed-upon communication protocols. The goal is to enable intelligent identification, tracking, and management of objects [27].

**Fig 2:**
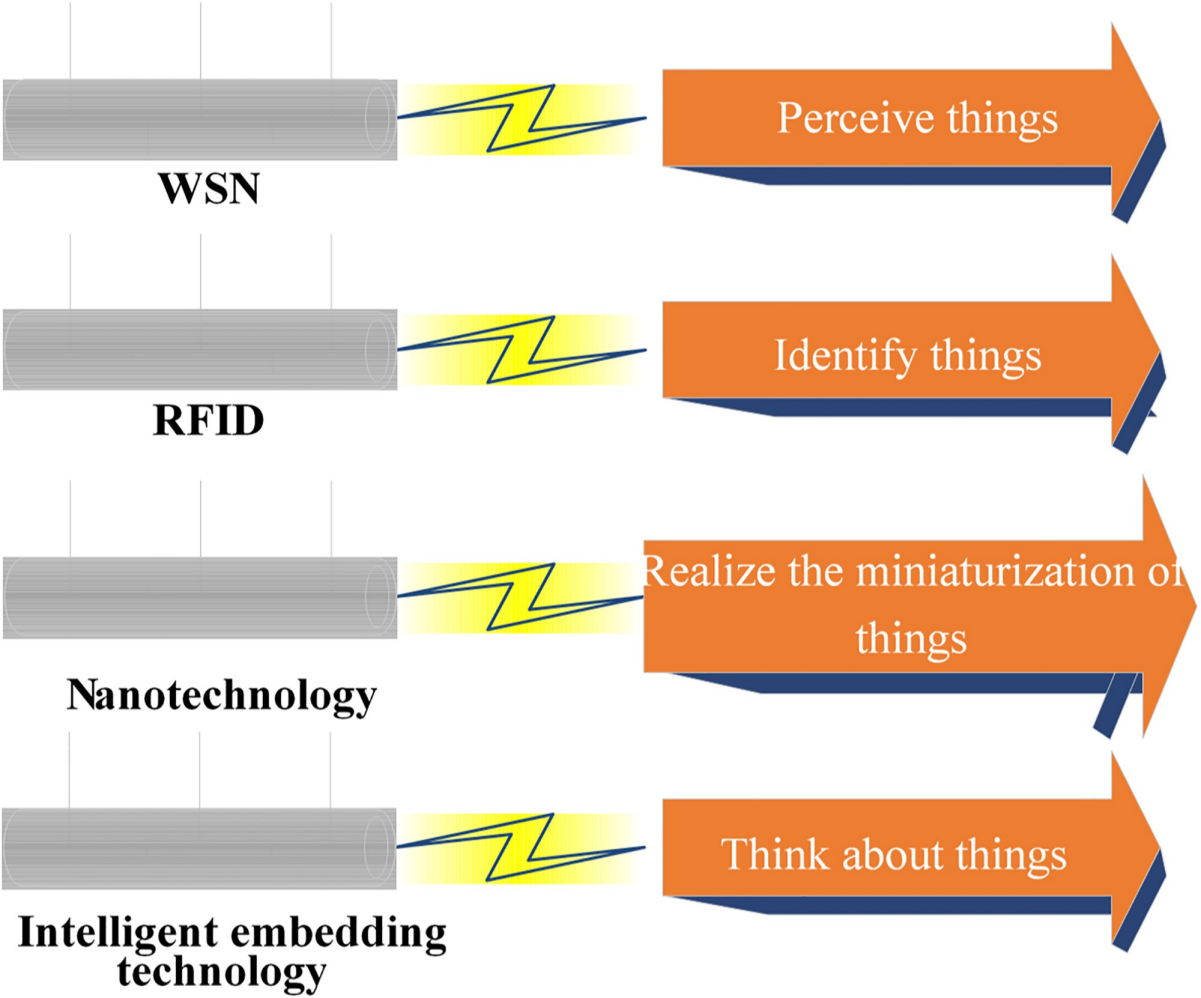
IoT key technologies.

IoT technology builds upon traditional Internet technology to create a network that facilitates information communication and exchange among a wide array of objects. It primarily encompasses the interconnection between Human to Human (H2H), Human to Thing (H2T), and Thing to Thing (T2T), effectively forming a network that links all things. IoT technology is still in its nascent stages, and researchers favor its broad development prospects. Four key technologies are underpinning IoT: sensor network, RFID, nano-technology and intelligent embedding technology [28]. Fig 2 illustrates their primary functions:

Key technologies of IoT include four aspects: WSN, RFID technology, nanotechnology, and intelligent embedding technology. WSN technology is primarily adopted to perceive things and form a monitoring network system through wireless communication. Each node cooperates with each other to conduct real-time monitoring, sense and collect monitoring data, and finally transmit the information to the user terminal. RFID technology primarily serves the function of identifying objects. By utilizing radio frequency signals, it enables wireless information transmission via spatial electromagnetic coupling, facilitating the identification of objects based on transmitted information. Nano-technology is mainly employed to realize the miniaturization of things. It enables the transition of IoT’s perception from the macro scale to the micro scale, ultimately achieving a comprehensive "perception of everything". Intelligent embedding technology is mainly adopted for the intellectualization of things, that is, thinking about things. It can achieve two-way communication and dialogue between objects and users or between objects and objects by implanting intelligent systems into objects to make them intelligent [29].

### 3.2 AI blockchain technology

AI is a multidisciplinary field that focuses on creating computer systems capable of simulating and emulating human intelligence. It encompasses various fields, including machine learning, natural language processing, computer vision, expert systems, and more. AI aims to equip computers with the capacity to replicate human cognitive processes, enabling them to exhibit traits like perception, comprehension, learning, reasoning, judgment, and decision-making. By analyzing extensive datasets and discerning patterns and trends within them, AI can make accurate predictions and informed decisions. Its applications span a wide array of domains, including intelligent assistants, autonomous driving, medical diagnosis, financial risk assessment, and more. The ongoing evolution of AI is a driving force behind technological and societal advancement, offering increased convenience and expanded possibilities for humanity. It requires people to strike a balance and regulate its development in accordance with ethics and societal values while promoting innovation.

AI is employed for the intelligent analysis of temperature data from power equipment. By learning from and comprehending extensive historical data, AI can identify unusual temperature patterns, thereby issuing alerts when temperatures reach specific threshold values. This alert mechanism assists operators in the early detection of potential overheating issues, enabling them to take appropriate measures to prevent equipment damage and fire hazards. AI technology endows the system with self-learning and adaptive capabilities, allowing intelligent assessment of equipment operational status based on collected data, and facilitating equipment fault detection and prevention. This significantly enhances the safety of power equipment operations. The integration of AI into wireless temperature monitoring systems elevates the system’s level of intelligence. AI not only aids in improving data processing and decision-making processes but also optimizes system performance and enhances the precision of data analysis. Consequently, it boosts the efficiency and reliability of the entire wireless temperature monitoring system in the realm of power engineering. In summary, AI plays a pivotal role in the application of AI-based blockchain technology combined with the IoT in wireless temperature monitoring systems for power engineering. It provides a crucial safeguard for the safe operation of power equipment.

The combination of AI and blockchain in wireless temperature measurement technology has brought innovative solutions to the power industry. This integration enables intelligent monitoring of power equipment while ensuring data security. AI’s role is to analyze temperature data from power equipment, providing real-time fault warnings and anomaly detection to prevent fires and equipment damage. Blockchain ensures data integrity, transparency, and data security and privacy protection, enabling trustworthy interactions and data sharing among multiple parties. This combination leverages the intelligent analysis capabilities of AI alongside the decentralized nature of blockchain, thereby enhancing the efficiency and reliability of wireless temperature measurement systems in the power industry, promoting safe operations, and fostering sustainable development.

As the IoT application level and grid intelligence continue to develop, a multitude of technologies and methods are emerging to address various power system challenges, becoming increasingly specialized. This section first analyzes the relevant concepts of smart blockchain technology. It then delves into the diverse functions that smart blockchain technology offers and analyzes its application in the power system, highlighting any existing issues. This analysis provides valuable insights for the subsequent establishment of wireless temperature measurement systems in electric power engineering.

**Fig 3:**
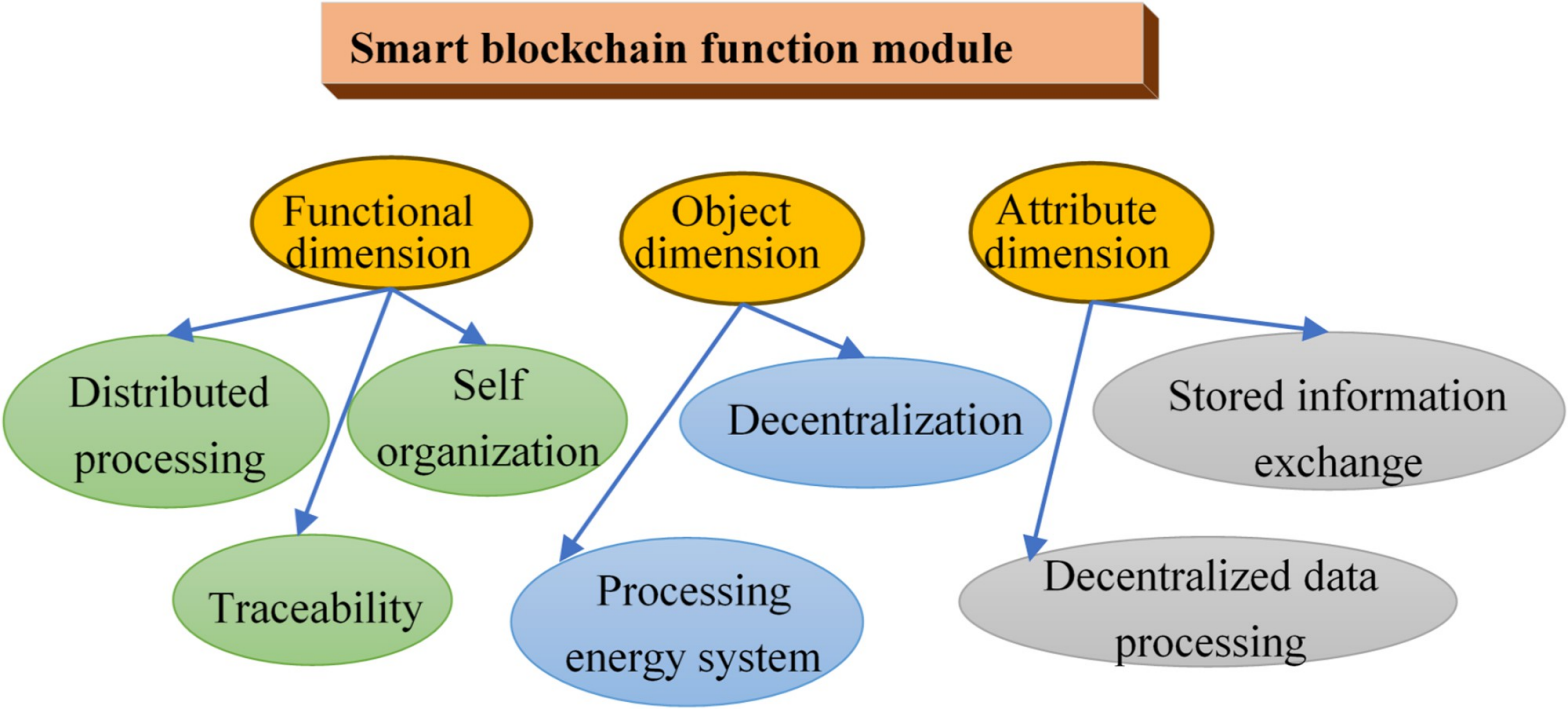
Function module of smart blockchain technology.

Blockchain, in this context, refers to a decentralized network where all nodes have equal status, eliminating the need for a central processor to exert control. The database of the smart blockchain boasts attributes of trustworthiness and high security. All information processing processes in the smart blockchain are open, fair and transparent [30]. Unlike the processing process of the traditional central processing unit (CPU), it can avoid the phenomenon of data information being stored and stacked in the CPU, and enhance data processing efficiency. Meanwhile, each node in the blockchain can process information and post-encode information. Relevant data information in the interconnection module can be collected, analyzed and authenticated, thereby enhancing the data mobility between adjacent modules. The smart blockchain can process data information in parallel, which improves the speed of information processing, reduces the pressure on the central server, and fully guarantees the security of data storage and analysis [31]. Fig 3 presents the three main functional modules of the technology.

The application of smart blockchain technology in the power system facilitates a direct connection between the electricity meter with the blockchain. The blockchain, as the basis, collects all power-related data, streamlining the conventional manual data transmission process and thereby enhancing data collection efficiency. The decentralized and tamper-proof attributes of smart blockchain technology ensure the security and transparency of power information. It leads to improved management efficacy within the power system, eliminating cumbersome intermediary steps and reducing losses and consumption during power transmission [32].

Smart blockchain can help build a safer and more efficient integrated IoT. Leveraging its big data and decentralized technologies, it empowers power grid regulation and the establishment of a decentralized power consumption model, which is more environmentally friendly. The microgrid based on blockchain, IoT and big data technology is currently employed in power system construction, with the characteristics of energy dispersion, load dispersion and power consumption nearby. It can supplement, optimize and replace the traditional power grid, making remote power supply a reality while offering a more reliable and secure operational environment for the power system [33].

To sum up, the integration of smart blockchain technology into the power system injects vitality into the entire power system, and improves the stability and security of the power system operation. Moreover, it can enhance the connection between various links, and provide a safe, convenient and efficient environment for power users to use electric energy. Looking forward, continued research into smart blockchain technology is imperative to address potential challenges that may emerge in the future development of power systems.

### 3.3 Wireless sensor temperature measurement system

**Fig 4:**
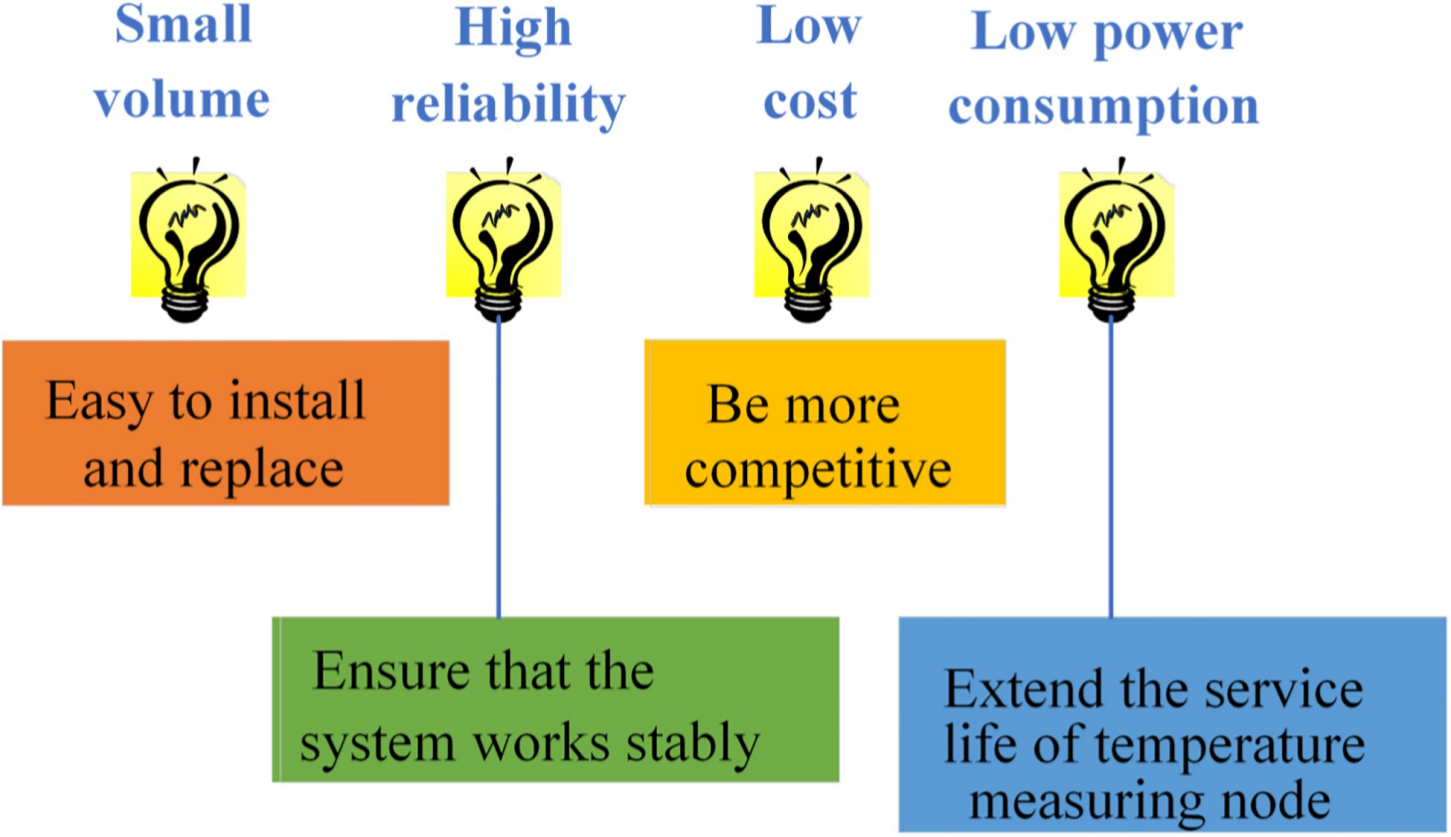
Performance of wireless sensor temperature measurement system.

As one of the key supporting technologies of IoT, WSN serves as an effective medium to integrate the physical and the information world. Despite being a well-acknowledged research focus within the information field, WSN continues to harbor several critical technologies and challenges that warrant exploration. The primary research contents are as follows: (1) Network topology control; (2) Network protocol; (3) Time synchronization; (4) Network security; (5) Data fusion; (6) Positioning technology; (7) Data management; (8) Embedded operating system [34]. This work primarily leverages WSN technology to study the temperature measurement system of electric power engineering. Fig 4 presents the performance requirements of the system design:

The volume of the temperature measurement system, particularly the temperature measurement nodes, should be minimized to reduce spatial occupancy, simplifying installation and replacement processes. In order to ensure normal and stable system operation and minimize temperature measurement errors, it requires reliable wireless communication between the transmitter and receiver. Given the potential for uncertain electromagnetic interference in the system environment, the system needs to exhibit a certain level of anti-interference performance. Each wireless temperature measurement system may contain hundreds of temperature measurement nodes. In commercial product design, the system’s cost should be comprehensively considered. While meeting system requirements, costs should be minimized to enhance competitiveness in the market. This is crucial because temperature acquisition points are powered by batteries, and reducing the power consumption of the temperature measurement nodes can extend their lifespan, reduce the frequency of battery replacements, and save on labor costs [35].

**Fig 5:**
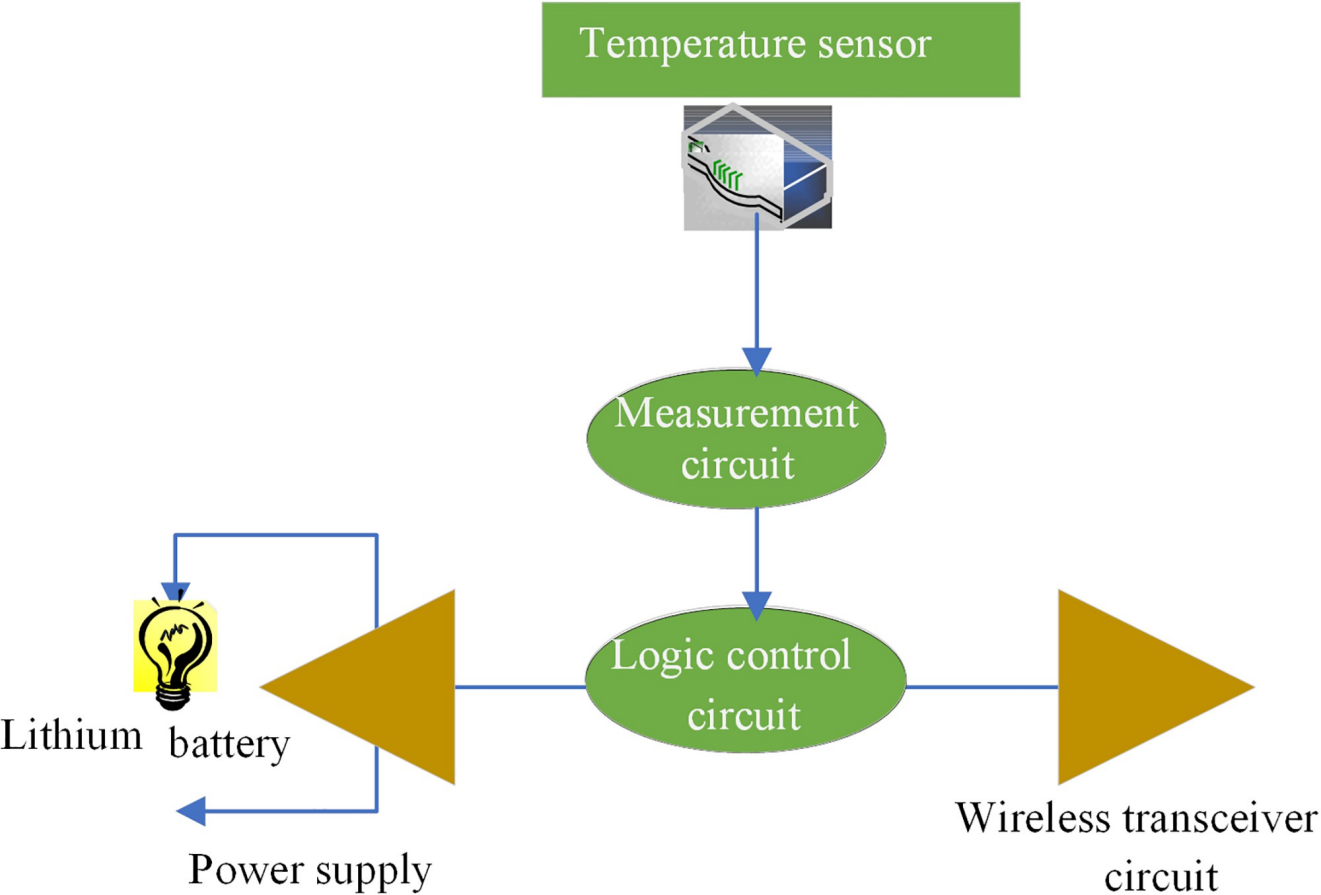
Structure of wireless temperature collector.

The wireless temperature collector is used for sensing and transmitting temperature signals, and is generally directly installed on the surface of the object to be measured, such as the exposed contacts of high-voltage lines and bus connection connectors. It comprises the temperature sensor, measurement circuit, logic control circuit, wireless transceiver circuit and power supply [36]. Fig 5 displays its connection structure:

The Finite Element Method (FEM) is employed to establish the distribution model of the power system temperature field, which can reflect the local temperature and temperature rise. FEM approximates continuous structures by dividing them into numerous small discrete elements and then solving analytical problems through mathematical modeling and calculations on these elements. In the temperature field distribution model, heat transfer issues within the electrical system can be discretized into a series of nodes and elements, with the temperature of each element determined by solving the heat conduction equation. Thereby, the model established by FEM is more accurate and reliable. The algorithmic steps for establishing a temperature field distribution model in the electric power system using FEM include: (1). Determine the geometric shape and material properties of the electric power system. (2). Divide the electric power system into numerous triangles or quadrilaterals. (3). Formulate the heat conduction equations for nodes and elements, and determine the heat conduction relationships between nodes based on material properties and boundary conditions. (4). Set initial conditions and boundary conditions, with an initial temperature of 25°C, the surface temperature of heat sinks in the electrical equipment at 80°C, and a maximum working temperature limit of 100°C for a critical component in the power system. One side of the power system is subject to radiative heat flux from the environment at 1000 watts. (5). Compute the temperature field distribution of nodes and elements by solving the discrete form of the heat conduction equations. The steady temperature field of the power system belongs to the two-dimensional steady heat conduction steady state. The temperature control equations of the area with a heat source and the area without a heat source are as follows [37, 38]:

$$\frac{d^{1}\theta }{dx^{2}}+\frac{d^{2}\theta }{dy^{2}}=\frac{\omega_{i}}{\lambda }$$ (1)

$$\frac{d^{2}\theta }{dx^{2}}+\frac{d^{2}\theta }{dy^{2}}=0$$ (2)

x and y are space coordinates, and λ denotes thermal conductivity. ω_i is the heat flux. The boundary conditions of any heat transfer problem can be classified into three types. The first type is the known boundary temperature, the second boundary condition is the known normal heat flux of the boundary, and the third type of boundary condition is the known convective heat transfer coefficient and fluid temperature. In this model, triangles are adopted to divide the grid. According to the linear interpolation function, the functional form in each plane triangle element is [39]:

$$J^{e}\left[\theta \right]=\iint \left[\frac{\lambda }{2}{\left(\frac{\partial \theta }{\partial x}\right)}^{2}+\frac{\lambda }{2}{\left(\frac{\partial \theta }{\partial y}\right)}^{2}-q_{v}\theta \right]dxdy-\int q_{0}\theta ds+\theta \int \alpha (\frac{1}{2}\theta^{2}-\theta_{f}\alpha \theta )ds$$ (3)

q represents the temperature value; ds is the thermal conductivity area; α is the temperature coefficient. In the establishment of the thermal conduction differential equation of the power system temperature field, the partial differential equation of solid thermal conduction with an internal heat source is as follows [40]:

$$\nabla ∙(k∙\nabla T)=-Q+c\frac{\partial T}{\partial t}$$ (4)

T is the temperature of each point in the power temperature field; k is the thermal conductivity; c is the specific heat capacity; Q is the generating heat of the heat source in unit volume; t is the time.

### 3.4 Wireless temperature measurement system for power engineering based on IoT combined with AI blockchain

**Fig 6:**
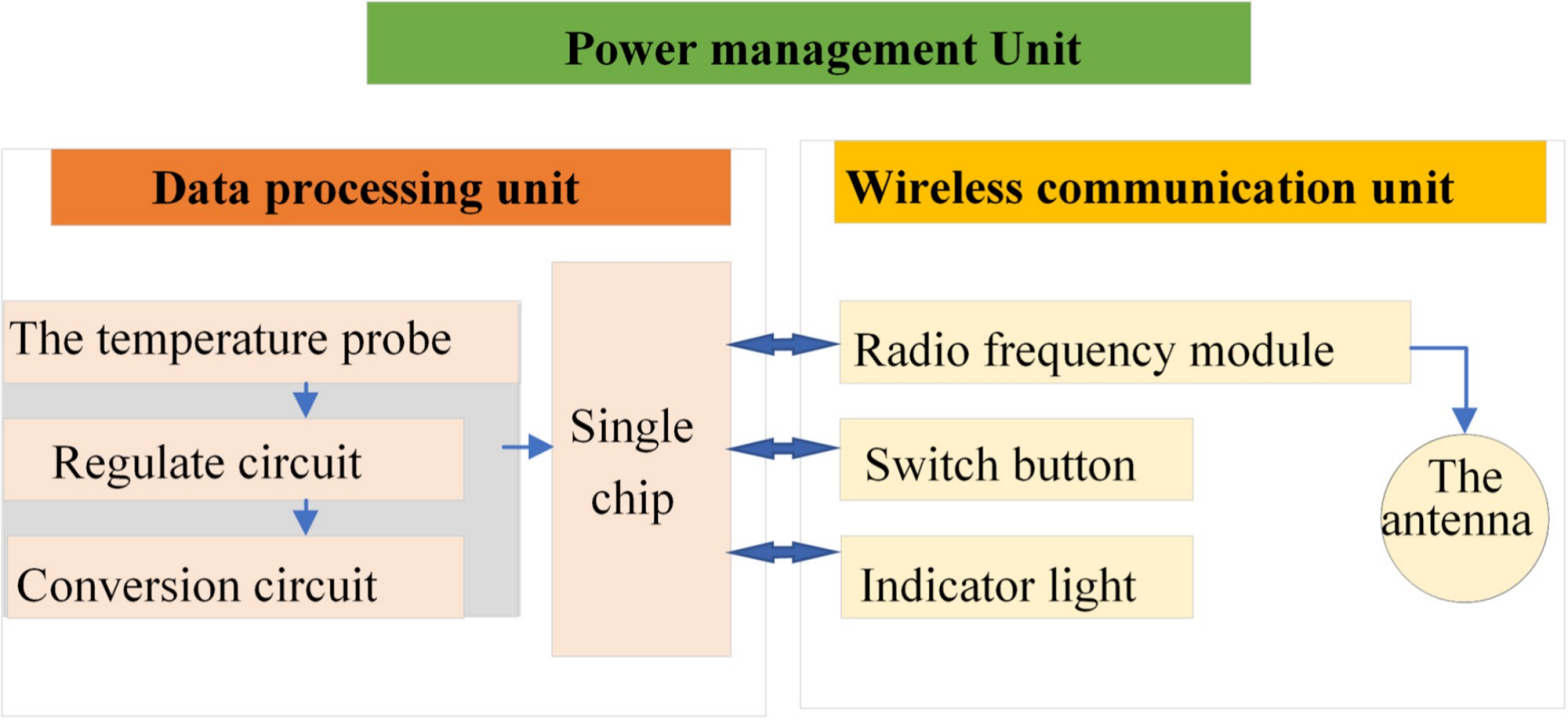
Principle of IoT-based wireless temperature measurement system of power.

The overall scheme of an IoT-based wireless temperature monitoring system for electric power involves the direct installation of temperature sensors onto electrical equipment to measure temperatures at specific equipment measuring points. Subsequently, these decentralized sensors are interconnected via wireless networks, establishing a temperature monitoring network for electrical equipment. This network incorporates relevant algorithms for the analysis and application of temperature data. The results provide a data basis for the power equipment maintenance department, enabling management personnel to promptly undertake repair and maintenance work on equipment operating at elevated temperatures. Besides, they can also comprehensively analyze the operation status of electrical equipment, formulate operational and maintenance strategies, reduce downtime and power outages, and prevent load loss [41]. Fig 6 displays the schematic diagram of the system:

The system utilizes IoT technology to collect real-time temperature data from power equipment through wireless sensors, which are then transmitted to a central processing unit for analysis and processing. Through AI technology, the system can intelligently analyze and predict temperature data, providing real-time fault warnings and anomaly detection to assist engineers in taking timely measures to prevent fires and equipment damage resulting from overheating. Additionally, blockchain technology plays a crucial role in this system. With blockchain’s decentralized and tamper resistance, the system ensures the security and trustworthiness of temperature data. All temperature data are recorded and stored in the blockchain format, making it tamper-proof and immune to alterations or deletions, thereby achieving data transparency and traceability. Moreover, blockchain technology facilitates trustworthy interactions and data sharing among multiple participants, promoting information security and collaboration.

**Fig 7:**
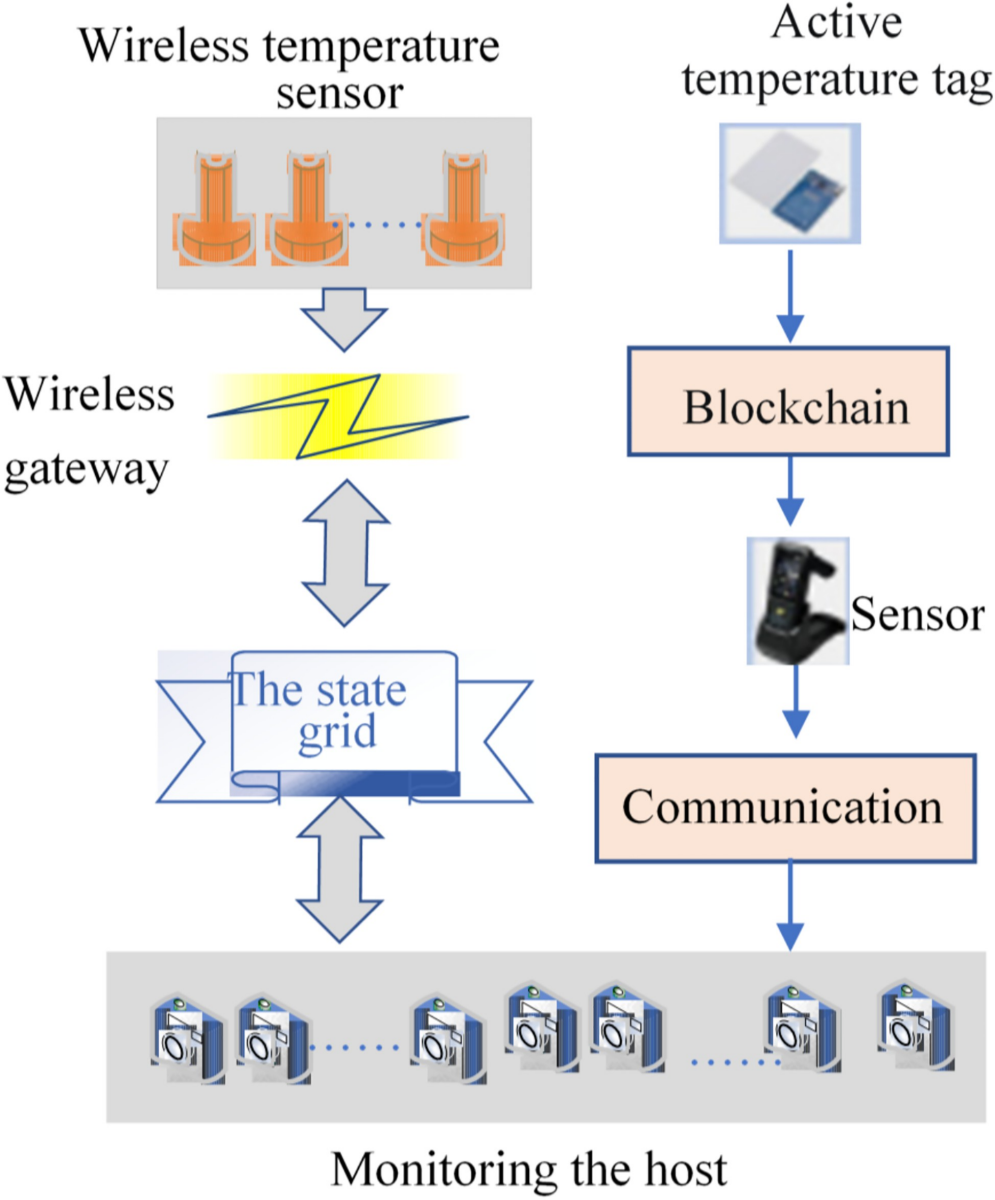
Temperature monitoring network of IoT wireless power equipment.

The IoT equipment monitoring technology standards, smart grid communication standards and IoT environment of power grid equipment are researched. Then, combined with IoT wireless sensor technology and smart grid equipment’s temperature measurement requirements, this work proposes an IoT-based wireless power equipment’s temperature monitoring network suitable for the power environment. The sensor node and coordinator node constitute the perception layer of IoT, the wireless network and Internet constitute the transmission layer of IoT, and the master controller and remote users constitute the application layer of IoT. The sensor node collects the temperature of the site and transmits it to the coordinator node through wireless. The coordinator node transmits the collected data to the master computer through the asynchronous transmission interface bus. The master controller analyzes, stores, preprocesses, and alarms the collected data. Remote users can monitor the status of devices in real time through the Internet [42]. Fig 7 displays its structure:

The wireless temperature monitoring network for power equipment serves as a supplementary system to ensure the seamless operation of power grid equipment, seamlessly integrating with the original power grid. The system comprises wireless temperature sensor nodes arranged at the measuring points, a data forwarding gateway and a monitoring host [43]. The standard protocol-based interface of the power grid terminal can communicate with the wireless power equipment’s temperature monitoring network, and collect the monitoring data to the monitoring host. The wireless temperature node wirelessly sends the data to the wireless gateway through the WSN protocol. The wireless gateway is connected to the national grid and forwards the data to the monitoring host at the grid terminal [44]. The figure illustrates the amalgamation of smart blockchain and IoT technologies, showcasing the utilization of various RFID tags and temperature collection, databases and blockchain. It perfectly creates a seamless connection with the temperature measurement system, eliminating additional system development and integration.

**Fig 8:**
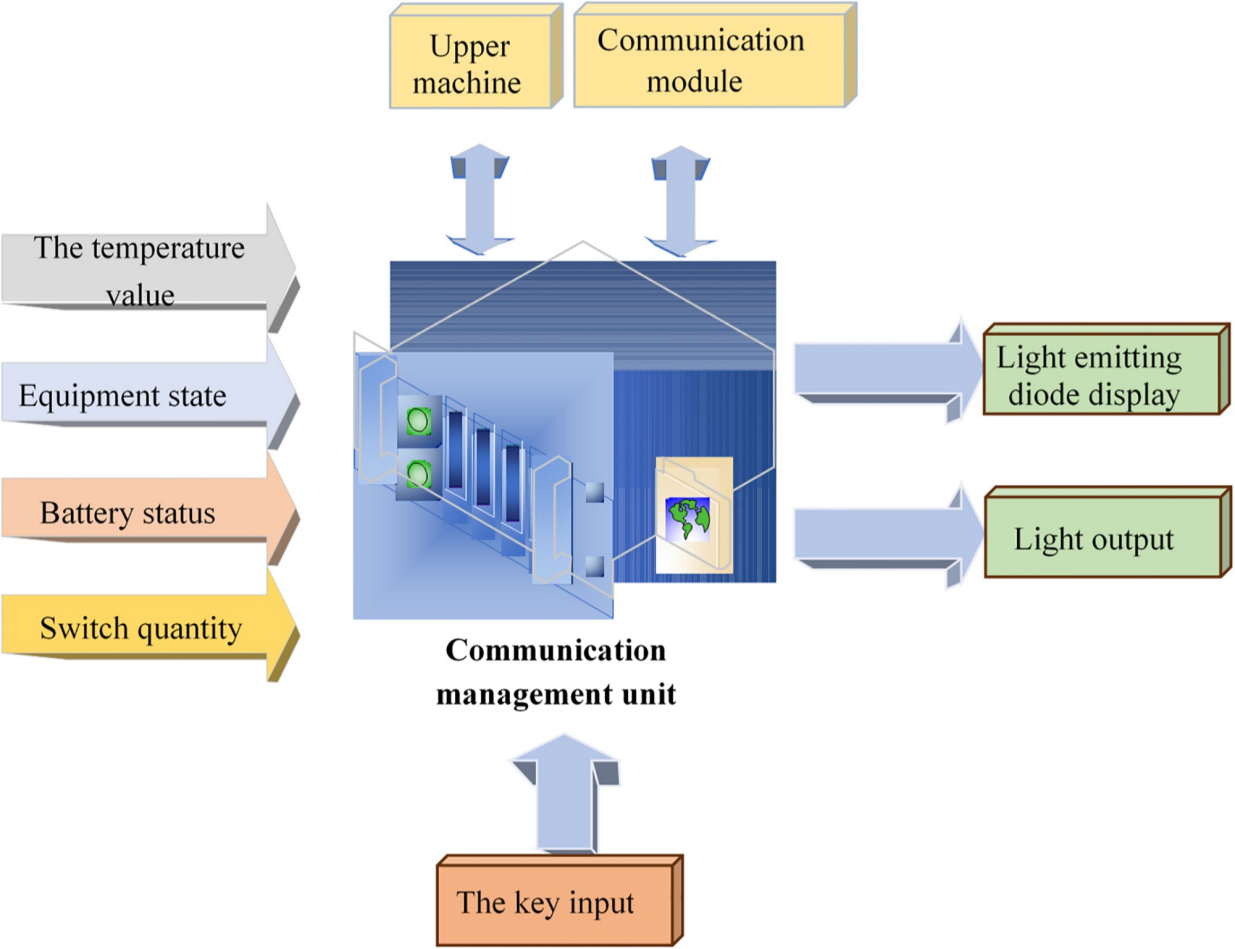
Control principle for the communication device.

The temperature collector’s primary responsibility is to collect the temperature value and transmit the data to the temperature-monitoring host through the wireless network. The temperature monitoring host processes and displays these data. When the temperature of the power equipment surpasses a predefined limit, the system automatically measures the temperature value and sends a high-temperature warning to the staff through the communication equipment. Then, related staff can promptly detect and perform maintenance to prevent accidents [45]. As the central management unit at the station end, the communication management device can realize the measurement control, communication management, data acquisition and processing of all temperature measurement units in the power distribution room. It can transmit the monitoring results and equipment status to the remote monitoring and data acquisition management system [46]. Fig 8 illustrates the control principle of its communication device:

The wireless temperature monitoring system in electric power engineering accomplishes continuous equipment tracking and over-temperature alarm functions, leading to significant savings in both manpower and material resources during power equipment inspections. It plays a positive role in improving the maintenance level of power equipment. The alarm status of communication equipment includes three types: overtemperature alarms of a single temperature measuring point, temperature rise alarms of a single temperature measuring point, and high-temperature alarms of temperature difference between equipment. When the sensor temperature T exceeds the set overtemperature alarm threshold, an alarm signal is generated immediately. However, the alarm signal returns when the sensor temperature T drops to the alarm temperature and the return coefficient. According to the calculation method of the maximum temperature rise allowed by the equipment, the temperature rise value T of the equipment within 1h per unit period is obtained. The actual temperature rise value of each temperature measuring point is compared with ΔT in real time to give an early warning when the equipment temperature rises due to defects but still does not reach the overtemperature alarm threshold [47].

Alarms are triggered for different data differences with different phases installed in the same equipment at the same installation location. Under normal conditions, the temperature between different phases at the same installation position of the same equipment should be relatively close. Under abnormal conditions, such as single-phase or two-phase faults, the temperature of the temperature measurement point of the fault phase will be higher than that of the normal phase [48]. The calculation for triggering the alarm is:

$$\left|T_{i}-\frac{T_{j}+T_{k}}{2}\right|\geq T_{\Delta up}$$ (5)

In (5), *T*_*i*_, *T*_*j*_ and *T*_*k*_ respectively represent a temperature in the three phases; *T*_Δ*up*_ is the set interphase temperature difference’s alarm value. According to the field operation experience, the over-temperature fault caused by equipment defects usually only occurs in the fault phase, while the normal phase is not affected much. Thereby, the interphase temperature difference alarm can be adopted to detect the single-phase or two-phase fault of the equipment promptly, and can even send the alarm of hidden trouble in advance [49].

The experiment project is divided into two parts: wireless temperature sensor installation, data networking and system debugging. There are 30 temperature sensors, 15 temperature measuring devices and 2 monitoring hosts in total. Table 1 outlines the main indexes of the wireless temperature sensor.

**Table 1:** Index of wireless temperature sensor.

Various indexes	Requirements	Various indexes	Requirements
Sensor service life	≥10 years	Operating frequency	430MHz
Temperature resistant environment	-25°C~125°C	System gateway accessible point	200
Maximum error of temperature measuring device	±2.0°C	Receiving sensitivity	-120dB
Temperature measurement accuracy	0.1°C	Overall dimensions	30×32×20(mm)
System measurement range	-25°C~125°C	Application scope	Power equipment, transformer station and so on

## 4. Results

**Fig 9:**
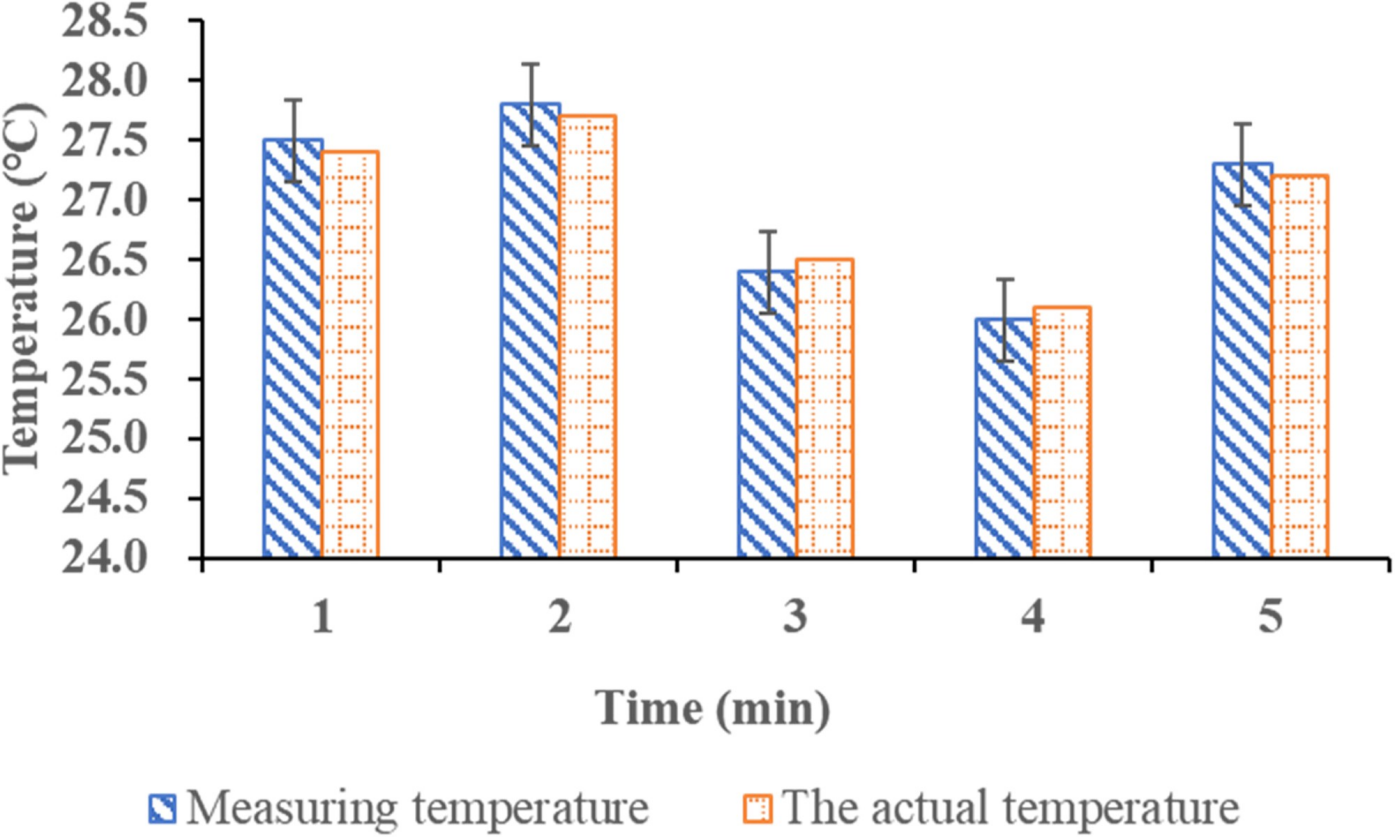
Comparison between test temperature and actual temperature.

Given the time-consuming and high cost of field inspection and monitoring, this experiment is arranged in the power laboratory. After the equipment is installed, the electric hair dryer is adopted to blow hot air one by one, and the difference between the displayed temperature and the actual temperature of the wireless sensor temperature measurement system is tested. Fig 9 displays the specific test:

Intermittent blowing is conducted within 5min to observe the temperature change and sensitivity. The results indicate that the overall temperature measurement value closely aligns with the actual temperature value, and the error range is within 0.1°C. It underscores the remarkably high measurement accuracy of the power’s wireless temperature measurement system supported by IoT, which can realize real-time monitoring of remote power equipment temperature. By analyzing the trend in monitored temperature values, potential high-temperature risks can be identified, leading to the timely dispatch of power maintenance personnel to assess the situation. It can effectively save the manpower consumed by traditional maintenance, and avoid the occurrence and loss of fire in electric power engineering.

**Fig 10:**
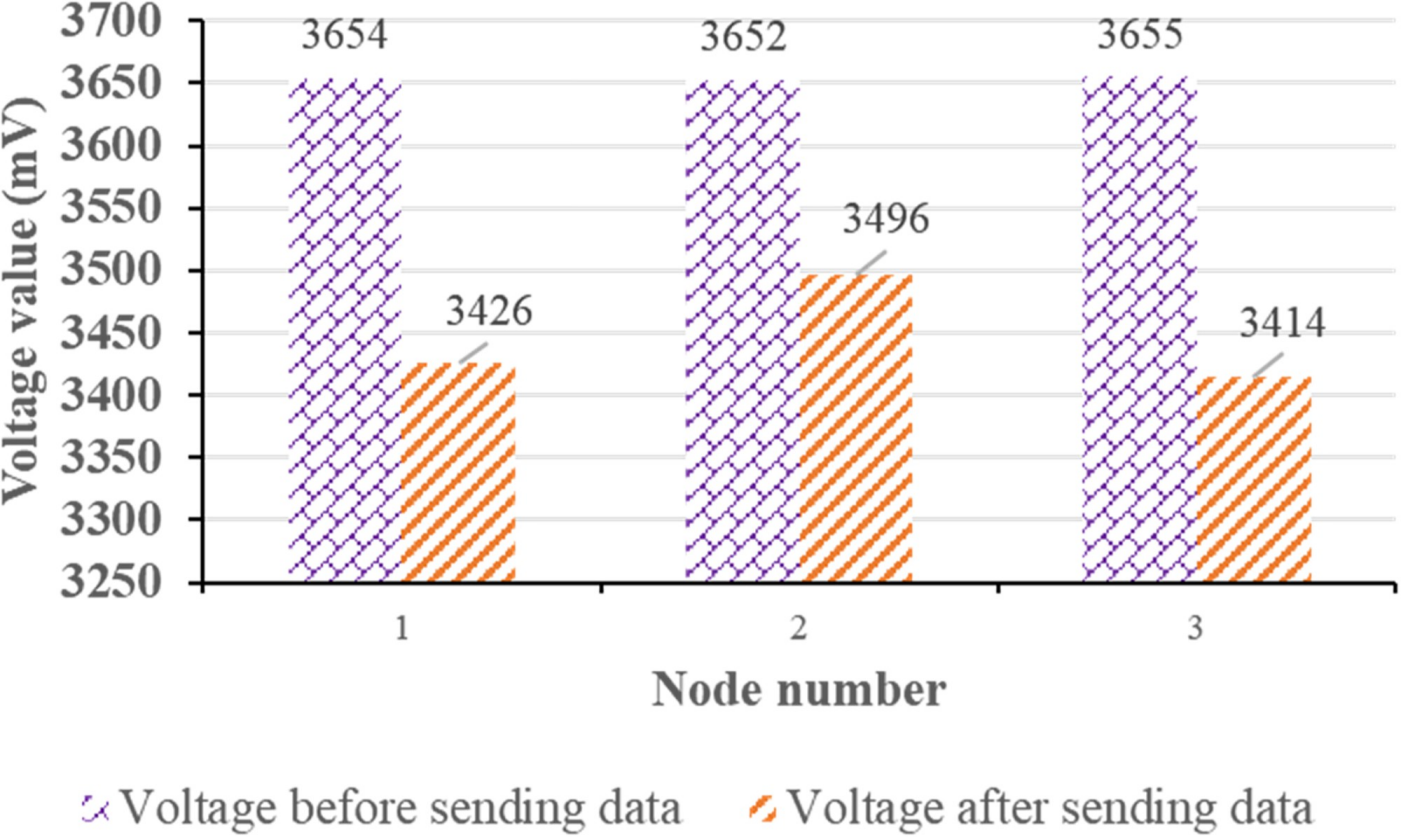
Battery life test.

The temperature measurement node studied is powered by a lithium cell. In order to mitigate concerns related to frequent maintenance due to battery life limitations, the nodes are designed with a strong emphasis on low power consumption. Besides, the battery service life test is carried out according to the actual use. Fig 10 displays the test results.

According to the above results, theoretical calculation and analysis are performed. When the temperature measurement node is optimized for low power consumption, there is a minimal increase in voltage, and it subsequently decreases after task completion. The primary power consumption during node sleep mode arises from sending, receiving, and sleep-related current consumption. In practical use, the node software design also makes the adaptive adjustment to the node transmission power, further extending the battery life.

After the temperature measurement of the power equipment is completed, the temperature out-of-limit alarm test shall be conducted. The temperature alarm value is set to 70°C. When the temperature value measured by the system reaches 70°C, the communication device will send an alarm signal to the power workstation staff. When the temperature drops, the alarm stops. The alarm monitoring test is performed on the equipment. Table 2 outlines the specific test conditions:

**Table 2:** Report of temperature alarm monitoring test.

Test items	Temperature measuring host	Monitoring computer
Over-limit value of temperature	Response	Receiving signal
The power loss of the device	Response	Receiving signal
Device communication failure	Response	Receiving signal

There are three experimental items in total: temperature overrun alarms, device power loss alarms, and device communication fault alarms. The test results demonstrate that when the alarm condition is reached, the temperature measurement host successfully sends out the alarm signal. The monitoring computer of the workstation also successfully receives the alarm signal and checks the real-time temperature change value in time. If the electric equipment is found to be in danger of fire, electric maintenance personnel will be sent to the site immediately for maintenance, effectively avoiding the threat of fire and minimizing irreparable losses. In this experiment, the power’s wireless temperature measurement system supported by IoT is tested and debugged. The wireless temperature monitoring system has passed various tests and can be connected to the network for operation and real-time monitoring of equipment temperature. The results affirm the system’s normal operation. The installation and successful operation of the power’s wireless temperature measurement system in this experiment verify the feasibility of integrating IoT with wireless sensor temperature measurement systems in electric power engineering. This accomplishment provides opportunities for the in-depth development and function improvement of the system.

**Fig 11:**
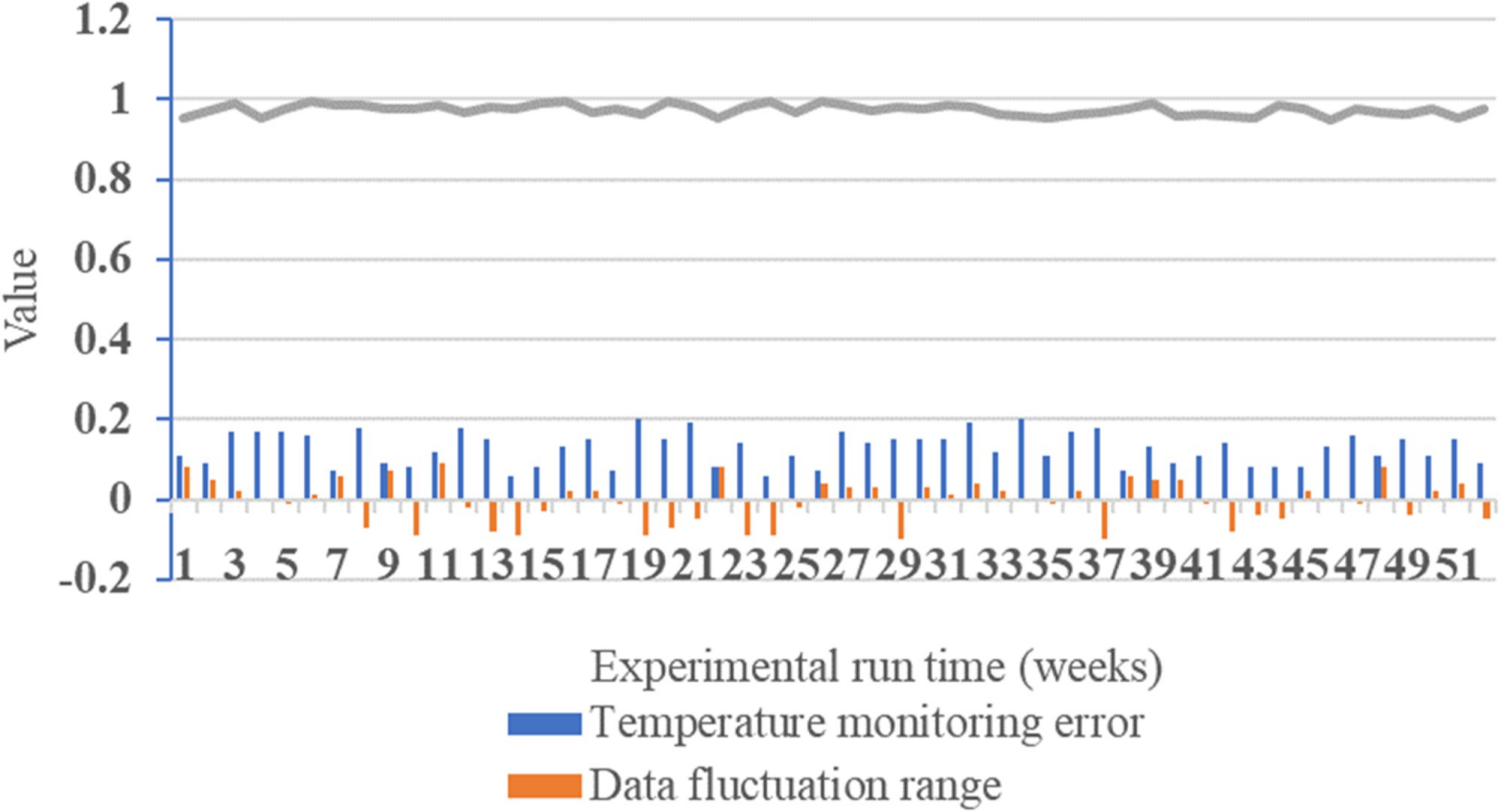
Test results for long-term stability.

The long-term stability, load variation impact, security, and scalability of the IoT wireless temperature monitoring network for power equipment in electric power environments are considered. Hence, in this experiment, the temperature monitoring errors, data fluctuation range, and temperature detection accuracy of this network are tested in continuous operation at high temperatures of 40°C to low temperatures of -10°C, under a relative humidity of 65%. Fig 11 illustrates the results.

**Fig 12:**
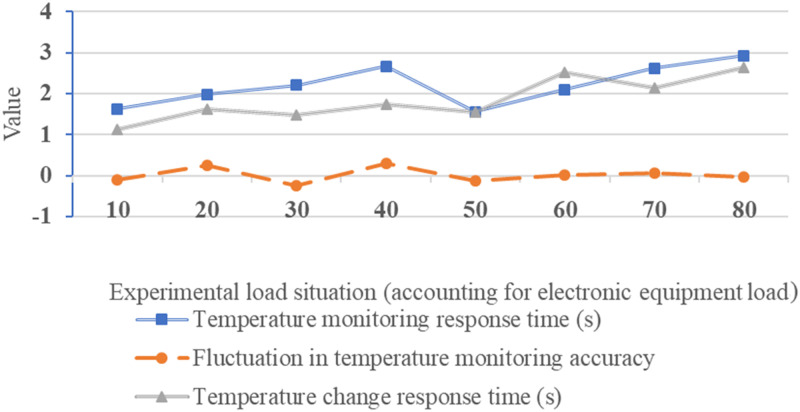
Test results for network load capacity.

Fig 11 displays the experimental data over a total duration of 52 weeks. Continuous operation from week 1 to week 52 validates the reliability of the long-term stability testing. Throughout various time intervals within the experiment, the temperature monitoring errors fluctuate within the range of 0 to 0.2, with a minimum error of -0.1 and a maximum error of 0.2. This indicates a high level of temperature monitoring accuracy and overall system stability. Data fluctuation remains between -0.1 and 0.1, signifying strong data stability with relatively small fluctuations. Throughout the experiment, temperature detection accuracy remains at or above 95%, reaching as high as 99.3% at times. This underscores the system’s sensitivity and precision in monitoring temperature variations, offering dependable data support. Fig 12 illustrates the results of the network’s load capacity test.

**Fig 13:**
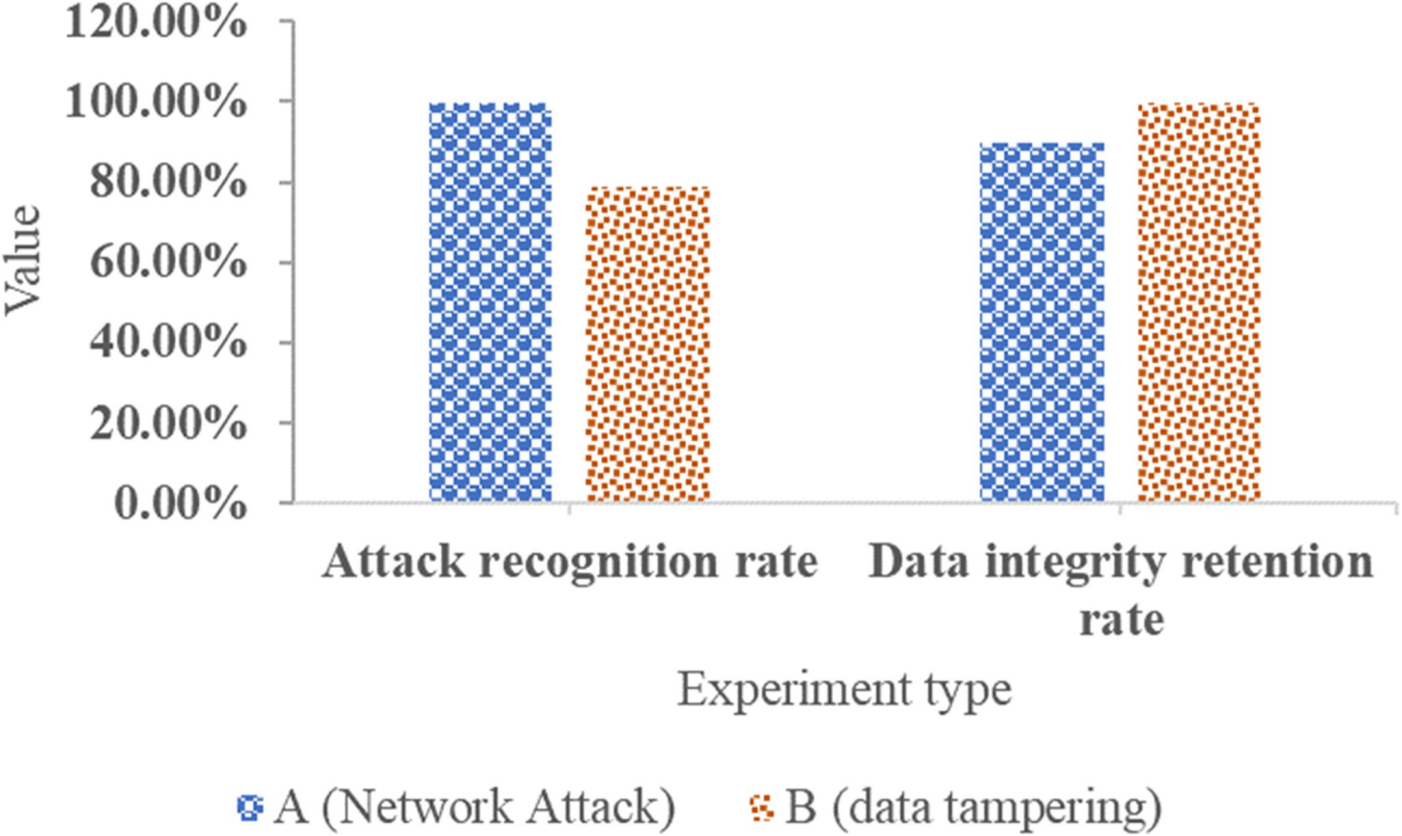
Test results for network security.

Fig 12 depicts the load testing conducted under load conditions ranging from 10% to 80%, ensuring performance assessment across various load scenarios. Under different load conditions, the temperature monitoring response times vary from 1.56s to 2.92s, indicating the system’s rapid response to temperature changes. Temperature monitoring accuracy fluctuations fall within the range of -0.25 to 0.3. The temperature change response time ranges from 1.13 seconds to 2.63 seconds, demonstrating the system’s strong temperature monitoring response capabilities and precision under various load conditions. It can adapt to fluctuations in the loads of different power equipment, ensuring timely and accurate temperature monitoring. Fig 13 presents the results of the network’s security testing:

Fig 13 displays the experiment types, which encompass network attacks (A) and data tampering (B), covering various aspects of network security, including network attack recognition and data integrity maintenance. The test results for network attacks (A) show a recognition rate of 99.50%, while the test results for data tampering (B) indicate a data integrity maintenance rate of 99.90%. These findings underscore the system’s high-level capabilities in attack recognition and data integrity preservation concerning network security. The system effectively shields itself from the threats of network attacks and data tampering. Table 3 illustrates the network scalability test results, which examine the system’s performance under various network load conditions, ranging from 50% to 100%. This ensures that the system possesses a robust network load-bearing capacity. As the network load increases, the data processing capacity expands from 100GB to 300GB. Alongside the increased network load and data processing capacity, the system’s response time gradually rises from 2.3s to 6.8s. This signifies the system’s ability to consistently deliver strong performance under varying network loads and data processing demands, ensuring stable operations in terms of network scalability.

**Table 3:** Test results for network scalability.

Network load (a percentage of electronic equipment load)	Data processing capacity	Response time (s)
50%	100GB	2.3
70%	150GB	3.1
80%	200GB	4.2
90%	250GB	5.5
100%	300GB	6.8

## 5. Discussion

When researching the design of a wireless temperature measurement system based on IoT, Jamroen et al. divided the system into three layers: temperature measurement workstation, temperature measurement terminal and temperature measurement node. Temperature data were then relayed to the monitoring software of the temperature measurement workstation through the temperature measurement node. Microcontroller and C language programming were used for experimental design. The experimental results suggested that the functions of the wireless temperature measurement system basically met the requirements. This system was user-friendly, characterized by a simple structure, and enabled real-time monitoring of power equipment temperatures [50].

In the design of a wireless temperature measurement system for power cables based on IoT technology, Feng et al. used Zigbee as the transmission protocol to collect and transmit temperature data in power cables. Additionally, they successfully amalgamated IoT with the smart grid, utilizing wireless sensors to construct a WSN and successfully realized the complete temperature data journey from collection to transmission to display [51]. Qasim et al. harnessed Raspberry Pi and IoT technology, coupled with SMS services and the global system for mobile communications, to create a real-time home security monitoring system [52]. Their research aimed to enhance home security by utilizing these technological means to monitor sensor data and send timely alerts regarding potential safety concerns. However, they did not verify the communication time, which might result in the inability to obtain real-time temperature data accurately. This suggested a certain limitation concerning ensuring real-time capabilities. Furthermore, due to the lack of data on system stability and long-term operation, the long-term reliability of the system also needed further validation and research. Feng et al. only considered the temperature measurement of power cables, without including the monitoring of other power equipment. Additionally, within complex power engineering environments, their system may encounter challenges such as interference and data transmission delays. While Qasim et al.’s research has achieved some success, it lacks in-depth data analysis. It prevents a comprehensive assessment of the system’s stability and real-time performance, particularly in terms of reliability during unexpected situations, which require further investigation and verification. In comparison with previous research, this work successfully incorporates IoT technology, AI, and blockchain technology into a wireless temperature measurement system for power engineering, enabling real-time monitoring and secure transmission of equipment temperature values. Temperature data are collected using IoT wireless sensors, with AI technology providing real-time analysis and anomaly detection. Furthermore, blockchain technology is applied to ensure data security and trustworthiness, preventing data tampering and unauthorized access. Experimental results demonstrate synchronized transmission time for temperature threshold alerts, ensuring simultaneous reception by monitoring equipment and real-time equipment temperature values. This work combines these technologies to elevate the intelligence of monitoring systems, ensuring both the accuracy and timeliness of equipment temperature monitoring, ultimately providing a safer and more reliable solution for the power industry.

## 6. Conclusions

The continuous advancements and maturity of AI and blockchain technology provide more powerful data analysis and processing capabilities for power engineering, further enhancing the intelligence level of wireless temperature monitoring systems. Through optimizing and learning AI algorithms, these systems can achieve more accurate fault prediction and anomaly detection, enabling proactive measures to prevent equipment overheating and fire hazards. Wireless sensor temperature measurement of power is a new way for people to measure temperature in power system, so the power’s wireless temperature measurement system based on IoT technology should be improved. First, the system should be well-designed. Based on a thorough understanding of the operating environment of power grid equipment, power grid technical specifications and current temperature measurement technology, this work proposes a temperature measurement system that meets the requirements of the power grid’s temperature measurement specifications. The system combines IoT wireless sensor technology to make the innovative application of the WSN in the power grid. It realizes remote temperature measurement, improves the reliability and practicability of the online monitoring system, and promotes the development of state detection technology of power engineering equipment. Meanwhile, experimental data clearly indicate that the overall temperature measurement values closely align with the actual temperature value, and the error range is within 0.1. It underscores the exceptionally high measurement accuracy of the IoT-supported power wireless temperature measurement system, which can realize real-time monitoring of the temperature of remote power equipment. There are still some research shortcomings. Future research efforts should focus on enhancing the transmission power of the temperature measurement nodes and optimizing the power supply mode for minimal power consumption to ensure the stable operation of nodes. Looking ahead, the evolution of AI-driven blockchain technology within IoT-based wireless temperature monitoring systems for power engineering will usher in further enhancements in system intelligence. These developments will fortify data security and usher in optimized management practices and efficient power system operations. This will bring greater convenience and development opportunities to the power engineering field and positively contribute to building sustainable and intelligent energy systems.
